# Correction: In pursuit of a cure: The plural therapeutic landscape of onchocerciasis-associated epilepsy in Cameroon–A mixed methods study

**DOI:** 10.1371/journal.pntd.0011114

**Published:** 2023-02-02

**Authors:** The PLOS Neglected Tropical Diseases Staff

There are formatting errors in the headings of the Results section. The headings should appear as follows:

3rd-level heading: Therapeutic landscape.

    4th-level heading: Biomedical care.

    4th-level heading: Indigenous care.

    4th-level heading: Faith healing.

3rd-level heading: Processes guiding therapeutic choice.

    4th-level heading: Socio-economic status.

    4th-level heading: Perceived effectiveness.

    4th-level heading: Drug shortages.

    4th-level heading: Distance and lack of infrastructure.

    4th-level heading: Patient follow-up.
