# Supplementary material for: In pursuit of a cure: The plural therapeutic landscape of onchocerciasis-associated epilepsy in Cameroon – A mixed methods study
Source: PLoS Negl Trop Dis. 2021 Feb 23;15(2):e0009206. doi: 10.1371/journal.pntd.0009206 (PMC7946181; doi:10.1371/journal.pntd.0009206)
Supplement: S1 Appendix — (PDF) [file pntd.0009206.s001.pdf]

S1 Appendix. Survey questionnaire.

IDENTIFYING INFORMATION

Date of visit: |__|__| / |__|__| / |__|__| (dd/mm/yy)
Village ID: |__|__|
Household ID: |__|__|
Participant ID: |__| (man=1, woman=2)

INDIVIDUAL INFORMATION

1. How long have you lived in this village?
    a. Since birth [*skip 2*]
    b. Less than one year
    c. One year
    d. More than one year [*go to 1.a.]*
    e. Other: ___________________________
    f. Don't know
    g. Refused

   1.a. [*If q1=d*] How many years have you lived here?
   Number (of years): ___________________________

2. Where were you born? [*Write down the region and country if not Cameroon*]
    a. Region/Country:____________________

FARMING

3. Do you farm or plant anything? |__| (YES=1, NO=2- If no, go to 11)

4. What do you plant or farm in all the rainy and dry seasons? *[Let the respondent answer, check all responses, then ask one by one for the unmentioned options].*
    a. Cocoa (cacao)
    b. Corn
    c. Peanut/Groundnut
    d. Cassava
    e. Macabo
    f. Taro
    g. Yam (Igname)
    h. Plantain/bananas
    i. Pistachios/Concombre (local terms)
    j. Palm
    k. Sugarcane
    l. Okra (Gombo in FR)
    m. Sweet potatoes
    n. Beans
    o. Vegetables (=leafs here) (légumes = feuilles in FR)
    p. Avocado tree
    q. Papaya
    r. Watermelon
    s. Orange/Mandarin/Grapefruit
    t. Lemon/lime
    u. Guava
    v. Pineapple
    w. Mango tree
    x. Plum tree
    y. Other *[Specify]*: ___________________
    z. Refused

5. Do you use any of the following products to fertilize farm soils? *[Multiple answers possible! Propose answer options. Make sure respondent does not effectively make use of products nor uses 'other' products than those shotlisted here before checking 'Don't use any product']*

1) Animal dung
2) Fertile soil
3) Animal feed
4) Food waste
5) Chemical fertilizers
6) Other *[Specify]*: ___________________________
7) Don't use any product
8) Don't know
9) Refused

6. Do you use any chemical pesticides? |__| (YES=1, NO=2, DK=3, REFUSED=4)

7. Are any of the farms you work on around your home? |__| (YES=1, NO=2, DK=3, REFUSED=4)

8. Are any of the farms you work on in the forest? |__| (YES=1, NO=2, DK=3, REFUSED=4)

9. Are any of the farms you work on in the savannah? (YES=1, NO=2, DK=3, REFUSED=4)

10. Are any of the farms you work on along the river bank or constant flowing stream? |__| (YES=1, NO=2, DK=3, REFUSED=4)

FISHING

11. Do you fish? |__| (YES=1, NO=2, REFUSED=3) – *If NO, go to 14.*

12. What method of fishing do you use?
[Multiple answers allowed - Let the respondent answer, then ask "do you use any other method?" each time respondent proposes an answer until he says "no"!]

a. With a rod, standing in the water
b. With a rod, NOT standing in the water
c. Block the waterflow and catch fish with baskets
d. With a net from a boat/pirogue
e. With a net, when the river is not too deep
f. Take fish from puddles with a machete
g. Put poison in the river
h. Other *[Specify]*: ____________________
i. Refused

13. What seasons do you fish in?
a. Big Dry season
b. Small rainy season
c. Small dry season
d. Big rainy season
e. All year round
f. Refused

HUNTING

14. Do you hunt? |__| (YES=1, NO=2, REFUSED=3)- *If NO, go to 16.*

15. What animals do you hunt? *[Multiple answers allowed - Let the respondent answer, then ask "do you hunt anything else?" each time respondent proposes an answer until he says "no"!]*
a. Porcupine
b. Snakes
c. Monkeys
d. Deer
e. Hare
f. Bush cat
g. Cutting grass / hedgehog
h. Rats
i. Don't know
j. Other *[Specify]*: _________________________
k. Refused

16. What is your main occupation? [*Only one answer!]*
   a. Farming
   b. Fishing
   c. Hunting
   d. Unable to work/Not currently working on anything/Unemployed
   e. Teaching
   f. Nursing care
   g. Traditional healing
   h. Housewife
   i. Student
   j. Other *[Specify]*: _________________________
   k. Refused

IVERMECTIN USE, PERCEPTIONS AND KNOWLEDGE

INTRO: We have been told that there is a program distributing Mectizan every year in some regions in Mbam.

17. Have you ever heard about mectizan? |__| (YES=1, NO=2, DK=3, REFUSED=4) [*Only one answer! If 'No', Inquire more to be sure respondent did not mis-interpret the question*] - *If NO, go to 27*

18. Do they also distribute Mectizan in this village? |__| (YES=1, NO=2, DK=3, REFUSED=4) – If NO, DK or REFUSED, *go to 20.*

19. When did they come last time?
   a. A year ago or less than a year ago (in the last 12 months)
   b. Between 1 and 3 years from now
   c. More than 3 years ago
   d. Other *[Specify]*: _________________________
   e. DK / don't remember
   f. Refused

20. In your personal opinion, is it useful to take it? [*Only one answer!]* (YES=1, NO=2, Sometimes=3, DK/not sure=4, refused=5)

INTRO: We know that there are sometimes unpleasant reactions when a person takes Mectizan.

21. Seen those reactions, do you think it is worth taking it? [*Only one answer!] (YES it's worth=1, NO it's not worth it=2, Sometimes=3, DK=4, Refused=5)*

22. Have you ever taken Mectizan? |__| (YES=1, NO=2, DK=3, REFUSED=4) (if YES, go to 23, if NO go to 22a, if DK or REFUSED go to 24)

   22a. Why not? *[Multiple answers possible! Don't probe]*
   a. Was away from the village at the time
   b. Afraid to take it because some people died taking it
   c. Afraid because it causes reactions (side-effects)
   d. I was told by doctors that I cannot take it
   e. The distributors never come to my house
   f. They ask us to pay money/ It's too expensive
   g. Was pregnant / I was breastfeeding
   h. It gives me rashes
   i. It makes my body swell
   j. I feel well, why should I take medicine when I feel well?
   k. No reason, I just don't want to take it
   l. I refused to take it [*enquire more please*]
   m. Other *[Specify]*: _________________________
   n. Don't know
   o. Refused to answer this question

Go to question 24!

23. Some people do not experience inconveniences but to others the medication causes some inconvenience. In your case, after taking Mectizan orally for the first time in your life, have you ever spent more than one year without taking Mectizan again? |__| (YES=1, NO=2, DK=3, REFUSED=4) → [*If NO or DK or REFUSED, go to 24*]

23a. Why? [*Multiple answers allowed! Don't probe*]

a. Was away from the village at the time
b. Afraid to take it because some people died taking it
c. Afraid because it causes reactions (side-effects)
d. I was told by doctors that I cannot take it
e. The distributors never come to my house
f. Sometimes the distributors don't come to my place
g. They ask us to pay money/ It's too expensive
h. Was pregnant/Was breastfeeding
i. It gives me rashes
j. It makes my body swell
k. When I feel well, why should I take medicine?
l. I refused to take it [*enquire more please*]
m. Other*[Specify]*: ________________________
n. Don't know
o. Refuse to answer this question

24. The last time that they came, how did they distribute the medication to you? [*Only one answer!*]

a. They came to my house (house-to-house)
b. I went to the health centre
c. I went to the chief's house
d. I went to the distributor's home
e. My relative picked up the medication for me
f. I wasn't there
g. I was here but I couldn't take it because… *[Specify]:* ________________________
h. Other *[Specify]*: ________________________
i. Don't know
j. Refused

25. In your personal opinion, what do you think Mectizan is used for? [*Let the respondent answer, then ask for each unmentioned options: "Do you think it can also be used against…(option)"*]

a. Black flies/moutmouts (YES / NO)
b. Worms (YES / NO)
c. Malaria (YES / NO)
d. Filaria (YES / NO)
e. Skin rashes (YES / NO)
f. Bumps (nodules) (YES / NO)
g. Eye/sight problem (YES / NO)
h. Onchocerciasis (YES / NO)
i. Mosquitos (YES / NO)
j. Other *[Specify]*: ________________________
k. I don't know
l. Refused

26. (*If q18=1*) How has Mectizan changed the health situation in this village? [*Suggest first 4 options*]

a. Didn't change
b. Improved
c. It's worse
d. Mixed feelings
e. Don't know
f. Other *[Specify]*: ________________________
g. Refused

EPILEPSY EXPERIENCE, PERCEPTIONS, KNOWLEDGE, ETIOLOGY

INTRO: We have been working in this region for more than a year and we have heard a lot about this problem of *epilepsy*. We are interested in knowing your personal opinion about it.

27. We are interested to know what different signs one can notice in someone who has epilepsy. If different people can have different signs, please tell us all signs that you can think of

[Let the respondent answer, then ask "anything else?" each time respondent proposes an answer until he says "no".]

a. Headache
b. Collapse/fall on the floor unexpectedly
c. Stop and become stiff
d. Body starts "shaking"
e. Has foam (bave/mousse) coming out of their mouth
f. Be confused/disoriented
g. Lose consciousness
h. Stare at nothing ("regarder dans le vide?")
i. Drop something they have in their hands (e.g. a bag, a spoon)
j. Be arrogant
k. Be stubborn
l. Be violent/aggressive
m. See/talk to things or people which/who are not there (hallucinations)
n. Hears voices
o. Head nods repeatedly
p. Speak an incomprehensible language
q. Scream
r. Run away
s. The person doesn't realise it just had a seizure
t. Starts acting like he's falling asleep when given food
u. Fatigue
v. Other *[Specify]*: _________________________
w. Don't know (Go to 29)
x. Refused (Go to 29)

28. Do you yourself have any of the signs you just mentioned? |__| (YES=1, NO=2, DK=3, REFUSED=4)

29. Do you think you have epilepsy?
a. Yes
b. NO (Go to 32)
c. I had it before but now I'm cured
d. I'm not sure / I don't know
e. Refused

30. Did a healthcare worker ever tell you that you have epilepsy? |__|(YES=1, NO=2, DK=3, REFUSED=4) → *[If NO or DK or REFUSED, go to 32]*

31. Where did you go to have treatment? *[Multiple answers allowed - Let the respondent answer, then ask "anywhere else?" each time respondent proposes an answer until he says "no"!*

a. Traditional healer
b. *Didn't seek any treatment*
c. Public Health centre
d. Missionary Health Centre
e. Lay Health Centre
f. Private practice (such as from retired nurse)
g. Hospital *(have an idea of the hospitals in the area)*
h. Church / through prayers
i. Directly from a pharmacy
j. Directly from mobile drugs sellers
k. I would ask an acquaintance who has an epilepsy victim how they treat it
l. I would treat it myself with herbs that I know

m. Other *[Specify]*: _________________________
n. Don't know
o. Refused

31a. (*If q31=a*) How do they treat traditionally there? [*Suggest options*]

a. Use of incantations / invokes spirits
b. Someone who sees the cause/ origin of the problem
c. Use of herbs/ barks
d. Other *[Specify]*: _________________________
e. Don't know
f. Refused

32. If one member of your family had epilepsy/local term, where would you go seek for a solution? [*Multiple answers allowed - Let the respondent answer, then ask "anywhere else?" each time respondent proposes an answer until he says "no"!*

a. Traditional healer
b. Public Health centre
c. Missionary Health Centre
d. Lay Health Centre
e. Private practice (such as from retired nurse)
f. Hospital *(have an idea of the hospitals in the area)*
g. Church / through prayers
h. Directly from a pharmacy
i. Directly from mobile drugs sellers
j. I would ask an acquaintance who has an epilepsy victim how they treat it
k. I would treat it myself with herbs that I know
l. Other *[Specify]*: _________________________
m. Don't know
n. Refused

32a. (*If q32=a*) How do they treat traditionally there? [*Suggest options*]

a. Use of incantations / invokes spirits
b. Someone who sees the cause/ origin of the problem
c. Use of herbs/ barks
d. Other *[Specify]*: _________________________
e. Don't know
f. Refused

33. Some people say that a person with epilepsy can contaminate another person, other people don't think so, what do you think? |__| (YES, a person can be infected by somebody with epilepsy =1, NO, it's not possible =2, DK=3, REFUSED=4)

34. In your opinion, what are the causes of epilepsy in your community? *[Let the respondent answer, then ask "anything else?" each time respondent proposes an answer until he says no]*

a. Malaria
b. Poor nutrition
c. Blackfly bite
d. Spirits/ancestors
e. Sharing food with someone with epilepsy
f. Sleeping next to someone with epilepsy
g. Having sex with someone with epilepsy
h. Bloodlines / intermarriage
i. Fall on head
j. neurological problem/ nerves problem (problème des nerfs)
k. Complications during pregnancy
l. Complications during childbirth
m. It's a physiological condition
n. God's will

o. Sorcery/witchcraft
g. Other *[Specify]*: ________________________
p. Don't know

INTRO: Since we've been working here, people sometimes tell us that epilepsy can be caused by sorcery.

35. In your opinion, can it be caused by sorcery? [*Only one answer!]*
a. YES
b. NO
c. Has said before that yes but now says no or don't know
d. Other *[Specify]*: ________________________
e. Don't know
f. REFUSED

36. Have you ever (now or in the past) known anyone who has epilepsy (even if they died)? |__| (YES=1, NO=2, DK=3, REFUSED=4) *[If NO, DK or REFUSED, go to 37]*

36a. Who are/were they? [*Multiple answers possible! Check the applicable relationship]*
a. Relative in household
b. Relative not neighbour, in this village
c. Relative neighbour in this village
d. Relative outside of this village
e. Close friend in household
f. Close friend not neighbour, in this village
g. Close friend neighbour in this village
h. Close friend outside of this village
i. Acquaintance (no close relation)
j. Acquaintance in household
k. Acquaintance not neighbour, in this village
l. Acquaintance, neighbour in this village
m. Acquaintance outside of this village
n. Other *[Specify]*: ________________________
o. Don't know
p. Refused

36b. What is/was the cause of epilepsy in this/these person(s) that you know/knew? [*Multiple answers possible - Check they give answers for the different persons mentioned in 36a]*
a. Sorcery
b. Genetics / family affected
c. Malaria
d. It's a disease like any other (=natural cause)
e. God's will
f. Didn't know
g. Other *[Specify]*: ________________________
h. Refused

37. In a case where epilepsy/maladie du tombe tombe is caused by sorcery, where do people go for treatment? [*Let the respondent answer, then ask "anywhere else?" each time respondent proposes an answer until he says "no"!]*
a. Traditional healer
b. Biomedical care (HC/hospital)
c. Church/through prayers
d. Buy medication (self-medication)
e. I would treat it myself with herbs that I know
f. It can't be cured/treated
g. It can't be caused by sorcery
h. Do nothing.
i. Other *[Specify]*: ________________________
j. Don't know
k. Refused

37a. (*If q37=a*) How do they treat traditionally there? [*Suggest options*]

a. Use of incantations / invokes spirits
b. Someone who sees the cause/ origin of the problem
c. Use of herbs/ barks
d. Other *[Specify]*: ____________________________
e. Don't know
f. Refused

37b. (*If q37=h*) Why do nothing?

a. It can't be cured
b. Other *[Specify]*: ____________________________
c. Don't know
d. Refused

38. As far as you've seen/hear/known, whether it's in the past or now, has epilepsy **always** been a problem in this village? [*Only one answer!]*

a. Yes, it has always been a problem in this village
b. No, **before** it was **more** of a problem, **now** it's **less** of a problem
c. No, **before** it was **less** of a problem, **now** it's **more** of a problem
d. No, it has never been a problem
e. Don't know
f. Other *[Specify]*: ____________________________
g. Refused

POTENTIAL RISK FACTORS

INTRO: We have seen that there are a lot of flying insects in this region and sometimes bother people. These are samples of insects we caught.

[Show the 3 samples with the insects (moutmout, mosquito and tse-tse fly)].

39. 39a. Can you tell me which sample is mosquito? [*Show all three samples at the same time - Only one answer!]*

a. Correctly identified
b. Didn't correctly identify
c. Other [Specify]: ____________________________
d. Can't see (Go to 41)
e. Refused

39b. Can you tell me which sample is tse-tse fly? [*Show all three samples at the same time - Only one answer!]*

a. Correctly identified
b. Didn't correctly identify
c. Other [*Specify*]: ____________________________
d. Refused

39c. Can you tell me which sample is moutmout? [*Show all three samples at the same time - Only one answer!]*

a. Correctly identified
b. Didn't correctly identify
c. Other [*Specify*]: ____________________________
d. Refused

40. 40a. Do you have this insect in your village? [*Point to mosquito]* |__| (1=YES, 2=NO, 3=DK, 4=Used to but now scarce, 5=Refused)

40b. Do you have this insect in your village? [*Point to tse-tse fly*] |__| (1=YES, 2=NO, 3=DK, 4=Used to but now scarce, 5=Refused)

40c. Do you have this insect in your village? [*Point to moutmout*] |__| (1=YES, 2=NO, 3=DK, 4=Used to but now scarce, 5=Refused)

Explain we would like to know more about the moutmouts or [USE LOCAL TERM].

41. If you think of all the places where moutmouts or [LOCAL TERM] bite you, during which activities do you get bitten most?

[Multiple answers possible - Let the respondent answer, then ask "anywhere else?" each time respondent proposes an answer until he says "no"!]

a. I have never been bitten
b. They are rare around here
c. By the river
d. At home
e. In the forest
f. At waterpoint while fetching water
g. Walking around the village
h. In the savannah
i. Everywhere
j. Other [*Specify*]: ______________________
k. Don't know
l. Refused

(*If q41=**c.***) During which activities at the river? [*Multiple options!]*

1) Fishing
2) *Farming*
3) Washing clothes
4) Swimming *(= leisure)*
5) Bathing by the riverside *(= hygiene)*
6) Walking around
7) Other [*Specify*]: ______________________
8) Don't know
9) Refused

➢ (*If answer = 2*) Which farms do you have there at the river? [*Multiple options!]*

a. Cocoa (cacao)
b. Corn
c. Peanut/Groundnut
d. Cassava
e. Macabo
f. Taro
g. Yam (Igname)
h. Plantain/bananas
i. Pistachios/Concombre (local terms)
j. Palm
k. Sugarcane
l. Okra (Gombo in FR)
m. Sweet potatoes
n. Beans
o. Vegetables (=leafs here) (légumes = feuilles in FR)
p. Avocado tree
q. Papaya
r. Watermelon
s. Orange/Mandarin/Grapefruit

t. Lemon/lime
u. Guava
v. Pineapple
w. Mango tree
x. Plum tree
y. Other *[Specify]*: ____________________
z. Refused

(*If q41=**d.***) Where at home do they bite? [*Multiple options!]*

1) Inside the house
2) Outside the house
3) On farms around the house
4) Other *[Specify]*: ____________________
5) Don't know
6) Refused

- (If answer=3) Which farms do you have around the house? [*Multiple options!]*

  a. Cocoa (cacao)
  b. Corn
  c. Peanut/Groundnut
  d. Cassava
  e. Macabo
  f. Taro
  g. Yam (Igname)
  h. Plantain/bananas
  i. Pistachios/Concombre (local terms)
  j. Palm
  k. Sugarcane
  l. Okra (Gombo in FR)
  m. Sweet potatoes
  n. Beans
  o. Vegetables (=leafs here) (légumes = feuilles in FR)
  p. Avocado tree
  q. Papaya
  r. Watermelon
  s. Orange/Mandarin/Grapefruit
  t. Lemon/lime
  u. Guava
  v. Pineapple
  w. Mango tree
  x. Plum tree
  y. Other *[Specify]*: ____________________
  z. Refused

(*If q41=**e.***) During which activities in the forest? [*Multiple options!]*

1) Hunting
2) Harvest wood
3) Cut down trees
4) Harvest fruits
5) Harvest herbs
6) Healing retreat
7) Farm
8) Other *[Specify]*: ____________________
9) Don't know
10) Refused

- *(If answer=7)* In which crops/plantation? [*Multiple options!]*

  a. Cocoa (cacao)
  b. Corn
  c. Peanut/Groundnut
  d. Cassava
  e. Macabo

f. Taro
g. Yam (Igname)
h. Plantain/bananas
i. Pistachios/Concombre (local terms)
j. Palm
k. Sugarcane
l. Okra (Gombo in FR)
m. Sweet potatoes
n. Beans
o. Vegetables (=leafs here) (légumes = feuilles in FR)
p. Avocado tree
q. Papaya
r. Watermelon
s. Orange/Mandarin/Grapefruit
t. Lemon/lime
u. Guava
v. Pineapple
w. Mango tree
x. Plum tree
y. Other *[Specify]*: ____________________
z. Refused

(*If q41=**h.***) During which activity in the savanah? [*Multiple options!]*

1) *Farming*
2) When taking care of the cattle
3) Other *[Specify]*: ____________________
4) Don't know
5) Refused

➢ (*If answer=1*) Which farms do you have there in savana? [*Multiple options!]*

a. Cocoa (cacao)
b. Corn
c. Peanut/Groundnut
d. Cassava
e. Macabo
f. Taro
g. Yam (Igname)
h. Plantain/bananas
i. Pistachios/Concombre (local terms)
j. Palm
k. Sugarcane
l. Okra (Gombo in FR)
m. Sweet potatoes
n. Beans
o. Vegetables (=leafs here) (légumes = feuilles in FR)
p. Avocado tree
q. Papaya
r. Watermelon
s. Orange/Mandarin/Grapefruit
t. Lemon/lime
u. Guava
v. Pineapple
w. Mango tree
x. Plum tree
y. Other *[Specify]*: ____________________
z. Refused

42. What do you do to avoid getting bitten by blackflies or [LOCAL TERM]? [*Multiple answers possible – Let the respondent answer, then ask "anything else?" each time respondent proposes an answer until he says "no"!*]

a. Nothing
b. cover myself (long sleeves, caps, socks)
c. apply Petrol
d. apply Lime Juice (lemon)
e. apply Palm Oil/red Oil…

f. apply pharmaceutical repellent
g. Mosquito net
h. Other*[Specify]*: ___________________
i. Don't know
j. Refused

43. Do you think that blackflies or [LOCAL TERM] can cause a disease? |__| (YES=1, NO=2, DK=3, REFUSED=4)

43a. (*If q43=YES*)
What disease can blackflies or [LOCAL TERM] cause? [Multiple answers possible - Let the respondent answer, then ask "anything else?" each time respondent proposes an answer until he says "no"!]

a. Onchocerciasis / filariosis
b. Filaria
c. Eye problem
d. River blindness
e. Malaria (*le Palu*)
f. Epilepsy
g. Skin rashes/itches
h. Nodules (FR:bosses)
i. Skin diseases
j. Body swelling
k. Sleeping sickness
l. Anaemia
m. Scabies
n. Other *[Specify]*: ___________________
o. Don't know
p. Refused

SOCIODEMOGRAPHIC INFORMATION AND EDUCATION

44. 44a. Age in completed years: |__|__| [*Enter 888 if age is not known!*]

44b. (*If 44a=888*) Year of birth: |__|__||__|__| [*Enter 888 if age is not given!*]

45. What is your tribe/ethnicity? [*Only one answer!*]

a. Bafia
b. Sanaga
c. Lemande
d. Bamileke
e. Yambassa
f. Yambeta
g. Bamenda
h. Bororo
i. Baneng
j. Eton
k. Other *[Specify]*: ___________________
l. Don't know
m. Refused

46. What is your mother's tribe/ethnicity? [*Only one answer!*]

a. Bafia
b. Sanaga
c. Lemande
d. Bamileke
e. Yambassa
f. Yambeta
g. Bamenda
h. Bororo
i. Baneng
j. Eton
k. Other *[Specify]*: ___________________

l. Don’t know
m. Refused

47. What is your father's tribe/ethnicity? [*Only one answer!*]
a. Bafia
b. Sanaga
c. Lemande
d. Bamileke
e. Yambassa
f. Yambeta
g. Bamenda
h. Bororo
i. Baneng
j. Eton
k. Other *[Specify]*: ___________________
l. Don’t know
m. Refused

48. What level of education have you attended? [*Only one answer!*]
a. Primary
b. First cycle secondary
c. Second cycle secondary
d. Higher education
e. No formal education
f. Refused

Record the location (GPS)
