# Supplementary material for: In pursuit of a cure: The plural therapeutic landscape of onchocerciasis-associated epilepsy in Cameroon – A mixed methods study
Source: PLoS Negl Trop Dis. 2021 Feb 23;15(2):e0009206. doi: 10.1371/journal.pntd.0009206 (PMC7946181; doi:10.1371/journal.pntd.0009206)
Supplement: S1 Table — For proportions, N = 1313. (PDF) [file pntd.0009206.s002.pdf]

S1 Table. Confidence intervals for weighted proportions, means and medians presented in the text*.
For proportions, N=1313

Variables	Estimate*	90% confidence interval*
**Socio-demographic characteristics of study participants**		
Migrants' duration of residence in the village, median number of years	8	7 - 9
**Activities**		
- Farming	1032 (75.3)	73.0 – 77.5
- Cacao farming	793 (64.9)	62.7 – 67.0
- Fishing	431 (27.0)	25.1 – 29.0
- Hunting	178 (13.0)	11.6 – 14.5
**Education**		
Primary school	629 (44.7)	42.4 – 47.0
First cycle secondary	459 (37.1)	34.8 – 39.4
**Median age survey participants**	35	35 – 37
**Perceived signs of epilepsy**		
Median number of signs	4	4 – 4
Unexpected falling or collapsing	1092 (82.3)	80.3 – 84.1
Shaking	917 (66.5)	64.2 – 68.7
**Perceived prevalence of epilepsy**		
Know someone with epilepsy	1212 (94.3)	92.5 – 95.6
Total self-reported epilepsy	64 (3.6)	3.0 – 4.5
Reported confirmation of epilepsy by a healthcare worker, among self-reported epilepsy	46 (71.1)	60.8 – 79.7
Mean age difference in years between (i) participants who self-reported having epilepsy and (ii) those who did not (i.e. the self-reported epilepsy group is younger)	6.9	5.0 – 8.9
Total male self-reported epilepsy	31 (3.3)	2.5 – 4.4
Total female self-reported epilepsy	33 (4.0)	3.0 – 5.3
Mean age difference for perceiving an aggravated epilepsy situation	11.1	9.4 – 12.7
**Perceived aetiology of epilepsy**		
Don't know the cause of epilepsy	570 (40.9)	38.6 – 43.3
Don't know but gave cause(s) anyway	39 (5.6)	4.2 – 7.3
Epilepsy is not contagious	997 (75.8)	73.7 – 77.8
**Comparison two questions about the cause of epilepsy:**		
(A) What is the cause of epilepsy? Answer: sorcery	574 (46.0)	43.6 – 48.4
(B) Can epilepsy be caused by sorcery? Answer: Yes	874 (84.0)	81.9 – 85.8
**Therapeutic landscape**		
Median number of answers in case *(i)*	2	1 – 2

Median number of answers in case *(ii)*	2	2 – 2
Median number of answers in case *(iii)*	1	1 – 1
Biomedical channels mentioned in case *(i)*	49 (74.5)	64.0 – 82.8
Biomedical channels mentioned in case *(ii)*	1075 (81.0)	79.0 – 82.9
Biomedical channels mentioned in case *(iii)*	659 (46.9)	44.5 – 49.3
**Knowledge about the aetiology of onchocerciasis and ivermectin**		
**Blackflies**		
Blackflies can cause a disease	1163 (88.5)	86.8 – 90.0
Blackflies can cause onchocerciasis/filariasis/filaria	493 (40.0)	37.6 – 42.5
Blackflies can cause malaria	364 (34.6)	32.3 – 37.0
Blackflies can cause skin rashes/itches	381 (31.4)	29.2 – 33.8
Blackflies can cause skin diseases	190 (15.3)	13.6 – 17.1
Blackflies can cause eye problems	142 (11.0)	9.6 – 12.6
Blackflies can cause scabies	109 (8.9)	7.5 – 10.5
Blackflies can cause epilepsy	17 (1.6)	1.0 – 2.4
**Preventive measures against blackfly biting**		
- Covering the body (with clothing)	1138 (86.1)	84.3 – 87.7
- Applying lime juice to the skin	146 (11.0)	9.6 – 12.6
- Applying petrol to the skin	129 (8.9)	7.7 – 10.2
**Knowledge about Mectizan**		
Heard of Mectizan	1294 (98.5)	97.6 – 99.1
Aware of Mectizan distribution	1235 (98.8)	97.8 – 99.4
**Perceived purposes of Mectizan**		
- Filaria	1135 (86.8)	84.5 – 88.7
- Skin rashes	951 (71.8)	69.0 – 74.4
- Blackflies	718 (51.7)	48.8 – 54.6
- Eye (sight) problems	857 (64.3)	61.4 – 67.1
- Bumps/nodules	661 (47.8)	44.9 – 50.8
- Worms	559 (42.3)	39.5 – 45.3
- Onchocerciasis	535 (39.8)	36.8 – 42.8
- Mosquitoes	532 (38.5)	35.7 – 41.4
**Community-directed treatment with ivermectin**		
**How was the drug distributed to you last time?**		
- To my house	710 (62.6)	60.5 – 64.6
- Went to community distributor's home	237 (12.9)	11.7 – 14.1
**Reported uninterrupted long-term uptake**		
- Spent more than one year without taking Mectizan since first uptake (= not continuous)	745 (61.6)	58.4 – 64.6
- Reported interrupted uptake – low prevalence villages	274 (56.5)	49.6 – 63.0
- Reported interrupted uptake – high prevalence villages	471 (67.4)	63.1 – 71.4
**Reasons for non-continuous yearly Mectizan uptake**		
- Away from the village at the time	171 (24.9)	22.1 – 27.9

- Distributors not visiting my house	153 (20.0)	17.5 – 22.8
- Pregnant/breastfeeding	141 (18.6)	16.2 – 21.3
- Afraid of side-effects	137 (18.4)	16.2 – 20.8
**Is it worthwhile to take Mectizan despite possible reactions?**		
- Yes	984 (76.0)	73.3 – 78.5
- No/sometimes	200 (15.6)	13.4 – 18.0
- Don’t know	108 (8.4)	6.9 – 11.4
**Impression of Mectizan**		
- Mectizan is useful	1136 (87.7)	85.5 – 89.6
- Mectizan has improved the health situation in the village	845 (68.6)	65.7 – 71.5

**Note:* Proportions are presented with unweighted frequencies as n (%), with confidence intervals likewise expressed as percentages. Differences between means are presented with the unweighted means in parentheses. Confidence intervals generalize to a finite population consisting of the nine sampled villages, while taking into account clustering in villages and households. Weights and finite population correction are applied using the estimated total number of households per village.
