# Supplementary material for: In pursuit of a cure: The plural therapeutic landscape of onchocerciasis-associated epilepsy in Cameroon – A mixed methods study
Source: PLoS Negl Trop Dis. 2021 Feb 23;15(2):e0009206. doi: 10.1371/journal.pntd.0009206 (PMC7946181; doi:10.1371/journal.pntd.0009206)
Supplement: S2 Table — (PDF) [file pntd.0009206.s003.pdf]

S2 Table. Characteristics (%) of the cross-sectional survey participants by village (quantitative strand).

Village	Bayom-en (N=124) n (%)	Kananga (N=157) n (%)	Nyamon-go N=166) n (%)	Bialan-guena (N=145) n (%)	Badissa (N=184) n (%)	Tcheckos (N=132) n (%)	Tchékané (N=150) n (%)	Ondou-ano (N=126) n (%)	Yebeko-lo (N=129) n (%)	Total (N=1313) n (%)[a]
**Age**										
16-30	43 (36)	36 (24)	60 (38)	57 (40)	53 (30)	47 (36)	51 (35)	34 (28)	54 (42)	**435 (34)**[a]
31-45	46 (38)	55 (36)	55 (34)	40 (28)	61 (34)	38 (29)	46 (32)	49 (40)	42 (33)	**432 (34)**[a]
46-60	22 (18)	37 (24)	31 (19)	27 (19)	50 (28)	26 (20)	34 (23)	24 (20)	21 (16)	**272 (21)**[a]
61-90	9 (8)	25 (16)	14 (9)	18 (13)	13 (7)	18 (14)	14 (10)	14 (12)	12 (9)	**137 (11)**[a]
Missing	4	4	6	3	7	3	5	5	0	**37**
**Education**										
No formal	13 (10)	9 (6)	15 (9)	3 (2)	7 (4)	8 (6)	7 (5)	9 (7)	5 (4)	**76 (6)**[a]
Primary	51 (41)	110 (70)	79 (48)	78 (54)	89 (49)	49 (37)	57 (38)	60 (48)	56 (44)	**629 (48)**[a]
Secondary - first cycle	39 (31)	29 (18)	58 (35)	51 (35)	69 (38)	56 (43)	69 (46)	43 (34)	45 (35)	**459 (35)**[a]
Secondary - second cycle	12 (10)	8 (5)	13 (8)	12 (8)	14 (8)	12 (9)	15 (10)	13 (10)	18 (14)	**117 (9)**[a]
Higher	9 (7)	1 (1)	1 (1)	1 (1)	3 (2)	6 (5)	2 (1)	0 (0)	3 (2)	**26 (2)**[a]
Missing	0	0	0	0	2	1	0	1	2	**6**
**Primary occupation**										
Farmer	76 (61)	137 (87)	126 (76)	126 (87)	155 (84)	102 (77)	114 (76)	112 (89)	85 (66)	**1033 (79)**[a]
Student	5 (4)	0 (0)	3 (2)	2 (1)	11 (6)	10 (8)	12 (8)	3 (2)	6 (5)	**52 (4)**[a]
Housewife	5 (4)	8 (5)	9 (5)	3 (2)	1 (1)	3 (2)	3 (2)	1 (1)	5 (4)	**38 (3)**[a]
Other	38 (31)	12 (8)	28 (17)	14 (10)	17 (9)	17 (13)	21 (14)	10 (8)	33 (26)	**190 (14)**[a]
**Region of origin**										
Centre Region	64 (57)	95 (92)	81 (64)	90 (85)	104 (87)	73 (79)	88 (88)	78 (79)	54 (67)	**727 (77)**[a]
Other regions	47 (42)	8 (8)	43 (34)	15 (14)	14 (12)	19 (21)	11 (11)	20 (20)	26 (32)	**203 (22)**[a]
Other country	1 (1)	0 (0)	3 (2)	1 (1)	1 (1)	0 (0)	1 (1)	1 (1)	1 (1)	**9 (1)**[a]
Missing	12	54	39	39	65	40	50	27	48	**374**
**Years living in village**										
< 1 year	25 (20)	4 (3)	20 (12)	12 (8)	8 (4)	7 (5)	10 (7)	16 (13)	28 (22)	**130 (10)**[a]
1-5 years	36 (29)	16 (10)	39 (23)	16 (11)	23 (13)	27 (21)	24 (16)	30 (24)	17 (13)	**228 (18)**[a]
6-10 years	23 (19)	18 (12)	23 (14)	23 (16)	18 (10)	21 (16)	10 (7)	17 (14)	8 (6)	**161 (12)**[a]
11-15 years	9 (7)	11 (7)	14 (8)	11 (8)	12 (7)	8 (6)	15 (10)	11 (9)	7 (5)	**98 (8)**[a]
16-20 years	6 (5)	8 (5)	13 (8)	10 (7)	21 (12)	7 (5)	8 (5)	9 (7)	5 (4)	**87 (7)**[a]
> 20 years	15 (12)	46 (30)	18 (11)	33 (23)	37 (21)	22 (17)	34 (23)	13 (10)	16 (13)	**234 (18)**[a]
Since birth	10 (8)	52 (34)	39 (23)	38 (27)	61 (34)	39 (30)	49 (33)	27 (22)	47 (37)	**362 (28)**[a]
Missing	0	2	0	2	4	1	0	3	1	**13**

[a] Unweighted percentages
