# Supplementary material for: In pursuit of a cure: The plural therapeutic landscape of onchocerciasis-associated epilepsy in Cameroon – A mixed methods study
Source: PLoS Negl Trop Dis. 2021 Feb 23;15(2):e0009206. doi: 10.1371/journal.pntd.0009206 (PMC7946181; doi:10.1371/journal.pntd.0009206)
Supplement: S3 Table — (PDF) [file pntd.0009206.s004.pdf]

S3 Table. Comparison of 'non-biomedical' treatment choices between 'low' and 'high' epilepsy prevalence villages in scenario *(ii)*.

	Village	N	Biomedical	Non-biomedical
**High Prevalence**	Bayomen	124	56.5%	43.5%
	Kananga	157	66.2%	33.8%
	Nyamongo	159	52.2%	47.8%
	Bialanguena	144	52.1%	47.9%
	Badissa	184	55.4%	44.6%
**Low Prevalence**	Tcheckos	132	49.2%	50.8%
	Tchékané	148	49.3%	50.7%
	Ondouano	125	52.0%	48.0%
	Yebekolo	128	78.1%	21.9%

Note: Here, we define 'non-biomedical treatment choices' as reporting at least 'Traditional healer' and/or 'Church/through prayers' and/or 'Self-treat with herbs/auto-medication' as treatment choices, regardless of also reporting other choices. 12 out of 1313 are missing values and are not used.

The weighted proportion of 'non-biomedical treatment choices' in low prevalence is 40.6%; high prevalence is 43.7%. Total is 42.0%.
