# Supplementary material for: In pursuit of a cure: The plural therapeutic landscape of onchocerciasis-associated epilepsy in Cameroon – A mixed methods study
Source: PLoS Negl Trop Dis. 2021 Feb 23;15(2):e0009206. doi: 10.1371/journal.pntd.0009206 (PMC7946181; doi:10.1371/journal.pntd.0009206)
Supplement: S4 Table — (PDF) [file pntd.0009206.s005.pdf]

S4 Table. Comparison of reported continuous ivermectin uptake between 'low' and 'high' epilepsy prevalence villages.

	Village	N	Continuous	Never or not continuous
**High Prevalence**	Bayomen	105	26.7%	73.3%
	Kananga	153	38.7%	61.3%
	Nyamongo	151	23.5%	76.5%
	Bialanguena	140	30.6%	69.4%
	Badissa	173	39.6%	60.4%
**Low Prevalence**	Tcheckos	121	40.8%	59.2%
	Tchékané	141	42.3%	57.7%
	Ondouano	112	39.3%	60.7%
	Yebekolo	113	37.4%	62.6%

Note: Here, we contrast continuous Mectizan use – those who reported not having gone more than a year without Mectizan after having started intake – versus those who have experienced periods of interruption or who never used Mectizan in the first place. 104 out of 1313 are missing values and were not used.

The weighted proportion for non-continuous Mectizan use in 'low' epilepsy prevalence villages is 56.5%; in 'high' prevalence villages is 67.4%, total is 61.6%.
