# Supplementary figures and images for: In pursuit of a cure: The plural therapeutic landscape of onchocerciasis-associated epilepsy in Cameroon – A mixed methods study
Source: PLoS Negl Trop Dis. 2021 Feb 23;15(2):e0009206. doi: 10.1371/journal.pntd.0009206 (PMC7946181; doi:10.1371/journal.pntd.0009206)

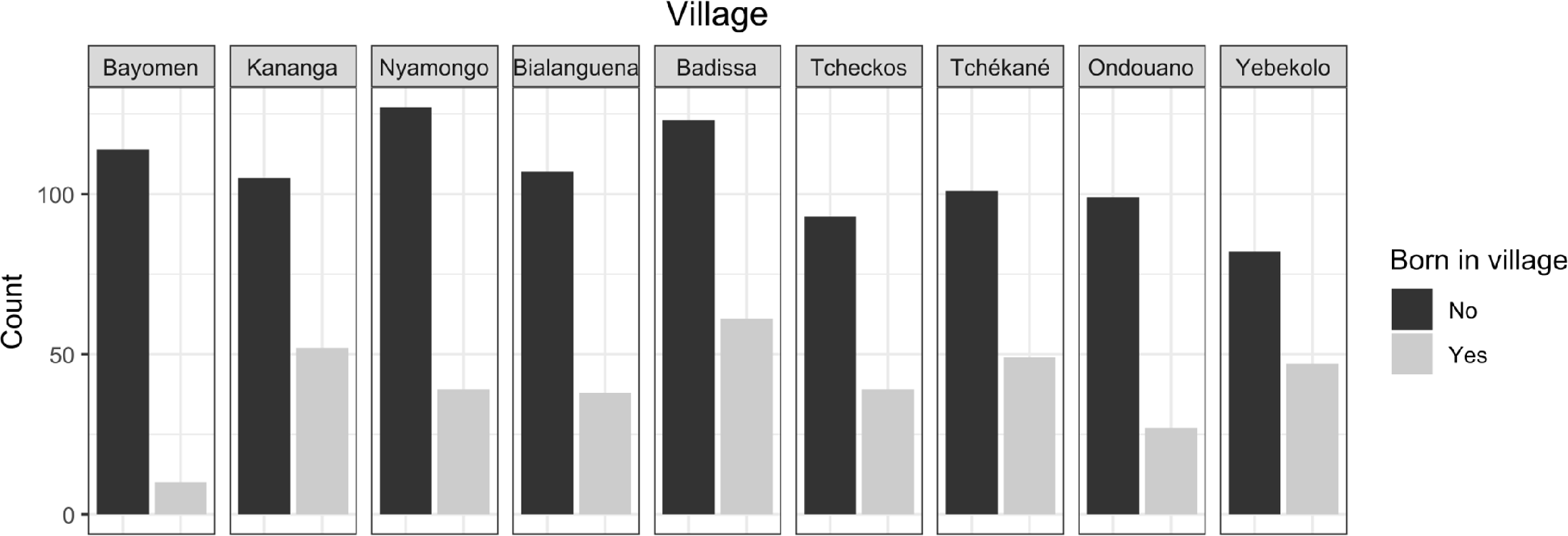

Supplement: S1 Fig — (TIF) [file pntd.0009206.s006.tif]

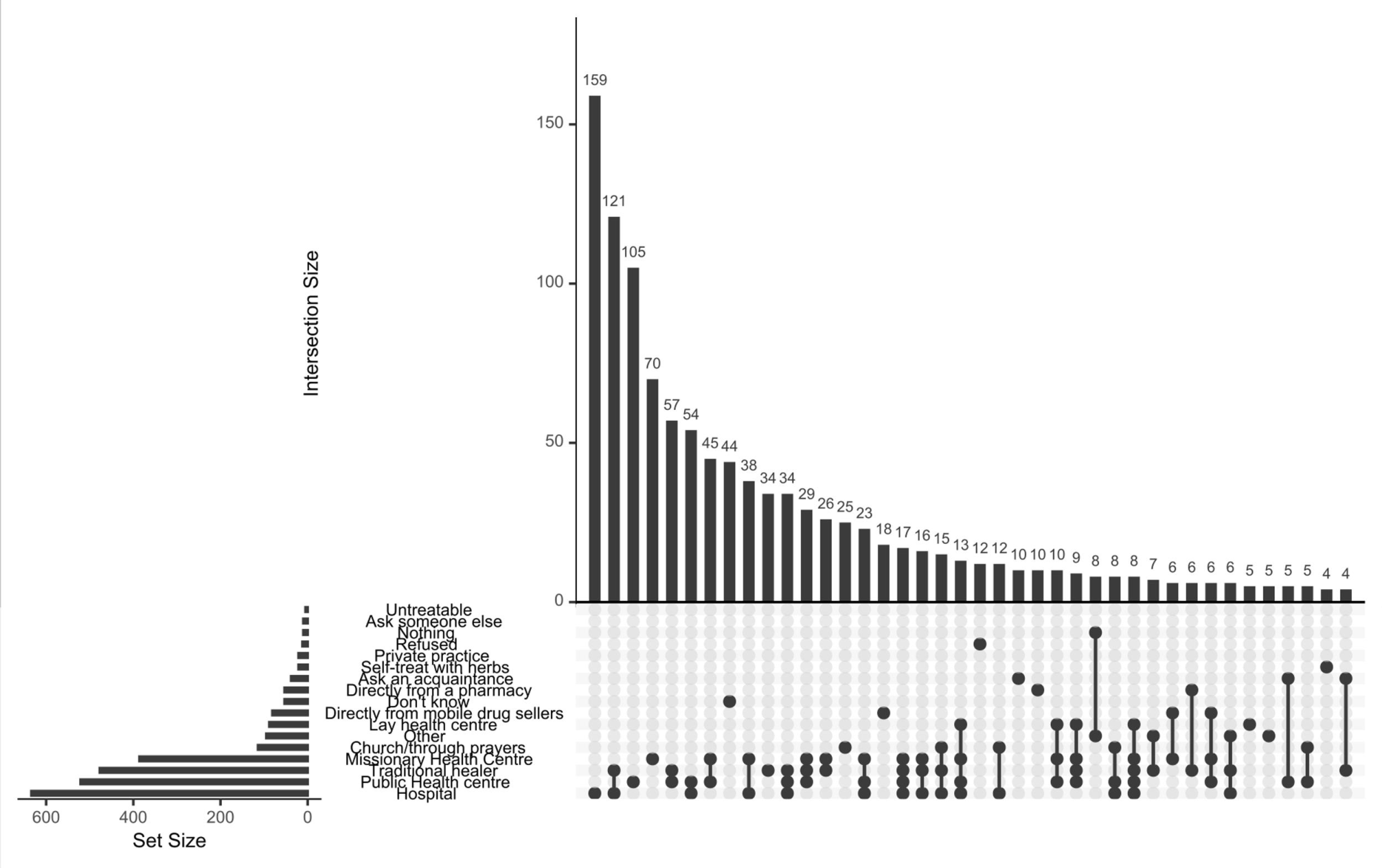

Supplement: S2 Fig — Note: The small graph on the left (Set Size) shows the individual frequencies for each answer category, while the graph on the right-side (Intersection Size) shows which unique combinations of answers were provided by the participants. As an illustration, the answer “hospital” was mentioned by 634 (49.1%) participants (cf. Set Size) and 159 (12.4%) participants mentioned it as the only treatment choice, while 475 (36.7%) mentioned “hospital” in combination with other options (cf. Intersection Size). (TIF) [file pntd.0009206.s007.tif]
