# Supplementary material for: In pursuit of a cure: The plural therapeutic landscape of onchocerciasis-associated epilepsy in Cameroon – A mixed methods study
Source: PLoS Negl Trop Dis. 2021 Feb 23;15(2):e0009206. doi: 10.1371/journal.pntd.0009206 (PMC7946181; doi:10.1371/journal.pntd.0009206)
Supplement: Attachment — Submitted filename: CMR HSB_Reply review 1_v1.1.pdf [file pntd.0009206.s009.pdf]

Cover Letter

Dear Lisa Dikomitis and Francesca Tamarozzi,

Thank you for giving us the opportunity to revise our manuscript. On behalf of the co-authors, I would like to express our appreciation for the thoughtful comments of the three reviewers. We gladly take this opportunity to clarify issues raised by the reviewers.

We have grouped the comments following the structure in which they were presented in the email and have responded to each of them immediately after each point. We replied to the reviewer's comments which were provided as an attachment to the email at the end of this document. In addition, we have addressed comments from the journal's technical checks including the one concerning the use of the term "unpublished data" (see comment n°17).

Together with this letter, we are resubmitting a clean and a "tracked changes" version of our work.

We hope we have been able to clarify the outstanding issues. We believe that the manuscript has much improved thanks to the reviewers' suggestions and hope that this revised version will now be considered for publication in PLOS Neglected Tropical Diseases.

Yours sincerely,

Maya Ronse

Responses to review comments for Manuscript PNTD-D-20-01745: "Epilepsy and onchocerciasis: the plural therapeutic landscape in pursuit of an epilepsy cure and experiences of 'community-directed treatment with ivermectin' in onchocerciasis-endemic Cameroon – a mixed methods study"

We thank the reviewers for their time and for their constructive comments. We have addressed their comments below and adapted the manuscript accordingly. When line numbers are mentioned in our response, they refer to the "tracked changes" version.

Methods

1. ***Reviewer #1****: The objectives of the study are clear, but the wording of the text makes it very difficult, if not impossible, to assess the appropriateness of the approach, methods, results and conclusions. This problem is common to the entire length of the text, so I will detail those problems together in "General Comments" section*

Response: We thank the reviewer for their comment. Considering the discrepancy between the comments received from different reviewers on this concrete issue, we have taken all comments into account in a concise way. We made the wording clearer in line with the reviewer's comments and addressed the detailed revisions in the corresponding "General Comments" section (n° 13).

2. ***Reviewer #2:*** *The research objectives are clear, and the methods used are appropriate. No concerns regarding the statistical analysis or ethical issues.*

Response: We thank the reviewer for their encouraging comment.

3. ***Reviewer #3:*** *Refer to attachment*

Response: We addressed the reviewer's comments which were provided as an attachment at the end of this document (cf. comment n°16).

Results

4. ***Reviewer #1****: Results are not clearly presented. Tables are sufficient and clear.*

Response: We have presented the results more clearly and structured them to increase clarity.

5. ***Reviewer #2****: Results are well presented in clear tables.*

Response: We thank the reviewer for this encouraging comment.

6. ***Reviewer #3****: Refer to attachment*

Conclusions

7. ***Reviewer #1****: Coclusions are mixed with methods and results. I miss some limitations, I'll explain in "General Commments"*

Among other reasons, this study is very interesting because it analyzes together very different types of factors, which are influencing in the actual results of implemented health programs.

Response: We provide further clarifications asked for in the "General Comments" section (comment n° 13), in which the reviewer further explained the comment above as indicated. We thank the reviewer for the encouraging comment.

8. ***Reviewer #2****: Conclusions are derived from the data. Discussions of the findings are relevant and shed some more light on the subject of onchocerciasis and epilepsy.*

Response: We thank the reviewer for their encouraging comments.

9. ***Reviewer #3****: Refer to attachment*

Editorial and Data Presentation Modifications

10. ***Reviewer #1****: Extension and structure are important concerns that should be modified.*

Response: We agree with the reviewer that the manuscript is relatively long, which is related to the fact that it tries to tie different interrelated analyses as was acknowledged by the reviewer in comments n° 7 and 13. These different results, however, need to be presented together since they impact the large and complex challenge of onchocerciasis-associated epilepsy, which from a community research perspective, need to be considered as two separate issues. We improved the structure of the results based on the reviewer's comments and made some passages more concise.

11. ***Reviewer #****2: I recommend acceptance after addressing a few minor comments.*

12. ***Reviewer #3****: Refer to attachment*

Summary and General Comments

13. **A**. ***Reviewer #1:*** *Authors, who demonstrate with so many and good bibliographic references of their own, that they are experts in the field, address an important health relevance problem. Among other reasons, this study is very interesting because it analyzes together very different types of factors, which are influencing in the actual results of implemented health programs.*

Response: We thank the reviewer for their detailed comments. We split them in several sub-sections in order to address each one specifically hereunder (A., B. etc.).

B. *One concerning issue, in my opinion, is the Tittle. The use of "epilepsy cure" in the title is not adequate. It is misleading. Onchocerciasis related epilepsy, even, fight against epilepsy, would be better options*

Response: We adapted the title and made it more concise: "*In pursuit of a cure: the plural therapeutic landscape of onchocerciasis-associated epilepsy in Cameroon – a mixed methods study*". The reference to a cure is linked to community perceptions in the health-seeking behaviour process. Despite the fact

that epilepsy can in many cases not be completely cured, and life-long symptomatic treatment is required, this reflects the hope for many affected persons that one day a cure will be found.

C. *However, I think my most important concern is with the length and order of the writing structure. I believe that authors should consider making a drastic reduction in the length of the text, avoiding repetitions, phrases and paragraphs that do not add information, and data that, although they have other types of interest, are not necessary for the analysis that the study presents. Besides, I find the article would improve and would be much more attractive and comprehensive to the reader adding order in its structure. These problems have made it very difficult, if not impossible, for me to assess the appropriateness of the approach, methods, results, and conclusions.*

Response: We shortened the text and improved the structure of the manuscript following the reviewer's suggestions. However, the public for which this manuscript was written is broad, including several disciplines and scientists interested in different methodologies and topics. We have therefore kept some findings from the qualitative research, which may be of less interest to some but a requirement for other experts. We improved the structure, have avoided repetitions and have shortened the text to allow the reviewer to assess the appropriateness of the approach, methods, results and conclusions.

D. *I can give some examples:*

Regarding structure:

-ABSTRACT, Methodology/Principal findings: I would like a clear and separate summary of Methodology, before talking about results. Later in the text, the authors use some more clarifying phrases that could be used. Even, it's not clear if they are presenting results or conclusions.

Response: The structure of the abstract follows journal instructions. Main results are presented in the "Methodology/Principal findings" section and the main conclusions are presented in the "Conclusions/Significance" section.

E. *-INTRODUCTION: Authors can consider to summarize, as background information, too many data that they present in Methods section. Those data are more explanations about why they decided to use those methods and make it difficult the understanding of the methods used. For instance, Methods are not the place to explain the background information: "Nodding Syndrome (a specific form of OAE)". There is a lot of information in the Methods-Study Setting section, even in the results section (for example, the different types of traditional health providers explanation) that belongs to the background information. Besides, issues related to Methods are in results, as "For absences or refusals, other household members of the same gender were sought out and, if any were available and willing, asked to participate."*

It would be convenient to extract from the whole text what is background, what are methods used, what are results and what are conclusions, and try to write them in their corresponding section.

Response: We thank the reviewer for these useful comments. We follow a mixed methods format on presenting methods and results. We reviewed the different sections, and where we found repetition, deleted to avoid repetition. We also moved certain parts that were indeed better placed in a different section, such as the part "Nodding Syndrome (a specific form of OAE)" which was moved to the "Introduction" section (lines 107-108) and the sentence "For absences or refusals, other household members of the same gender were sought out and, if any were available and willing, asked to participate" which was moved to the "Methods" section (lines 261-263). Regarding the suggestion to move the different types of traditional health providers described in the "Results" section to a background information section, we however kindly disagree since these constitute actual research

findings. We cannot assume the general categorization of traditional healers applies to the whole "African" context, which is why we researched these categories in this specific context.

F. *I needed to print out the text to understand under which heading some sub-headings were included. In spite of this, I was not able to understand the structure. In "375 Perceived aetiology of epilepsy". It this a new epigraph? Should it be written in bolds as the previous epigraphs?, regarding "Natural" "Mistical" "Worms" and "Diet", are they sub epigraphs of "Perceived aetiology of epilepsy"? I recommend making it clear with numbers or an organized structure of type of letters.*

Same comments regarding "447 Therapeutic landscape", "495 Processes guiding therapeutic choice" "587 Community-directed treatment with ivermectin"

Why are "287 Ethical considerations" in Methods? If not, please, made it clear.

Response: We agree that considering the complexity of the research topic and the use of mixed methods, the structure might benefit from clearer subtitles and maybe some numbering of the titles. However, we followed the journal instructions in terms of paper structure and format to be consistent with the guidelines. If the editors allow for changes at this level, we proposed a few changes in attempt to provide more clarity to the overall structure (see manuscript). Nevertheless, if numbering in titles are not allowed, they can be removed again. Here is an overview of the heading hierarchy proposed with numbering suggestions between brackets:

Level 1 heading
Level 2 heading (1.)
Level 3 heading (1.1.)
Level 4 heading

We hereby hope the structure has now become clearer. Finally, concerning the "Ethical considerations" section, we followed a structure and format commonly seen in PLOS NTD articles where the ethical considerations are described in the methods section, which is the reason why we followed this structure.

E. *Regarding extension:*

From lines 78-103, text could be considerably shortened. The article's aim is not about epilepsy, but its relationship with Onchocerciasis. Authors should consider keeping just those data related with the topic of interest of this paper.

448-454: It would remove or considerably reduce some very complex sentences, and/or those that do not provide new information, because is already reflected in the text: "The complexity of the various illness interpretations within the vast and therapeutic landscape varied in our study setting led to health seeking itineraries that were equally complex, combining all manner of options in the often-desperate quest for a cure." "Regardless of its perceived aetiology, epilepsy was generally said to be incurable, with only symptom suppression as a way of managing the illness. Nevertheless, a few stories circulated of a complete cure after indigenous treatment or faith healing. Alternating and combining several types of care in the process of searching for a diagnosis and treatment were common."

397-495: It is very interesting to read about mystical causes and worms, with their anecdotes and literal stories included. But I consider that the extension is excessive and that it could be reduced without losing the understanding of the type of beliefs found. It is possible that these explanations could constitute another type of article, very interesting, but in this case it does not provide neccesary data to support the conclusions.

306-309. Introduction to results may be not necessary because the different data collected are already, or should be, in the methods.

For the entire text, I recommend analyzing the statements that are repeated several times in the different sections and avoiding them whenever possible.

Response: Several of the reviewer's comments hereabove are about reducing repetition and length of text. We reworked the different mentioned sections and made the entire manuscript more concise according to these suggestions. The article's aim is to provide insights into the health seeking behaviour for epilepsy as well as into the adherence to ivermectin mass drug administration, distributed through the on-going CDTI programme. At community level, and in relation to people's perceptions and knowledge, it is impossible to distinguish whether an epilepsy case is associated to onchocerciasis as the symptoms are the same. Community members can therefore not distinguish, nor refer, to epilepsy in general versus OAE. It is therefore important that the readers (who are not all experts in epilepsy) understand epilepsy broadly in order to understand the results.
Please see lines 523-541 for the changes related to initial lines 448-454. Regarding the suggestions for lines 397-495, this is part of the health seeking behaviour. This level of details is important for the reader as it illustrates the combination of both mystical and natural causations, and supports the conclusion regarding the plural therapeutic landscape for epilepsy in the study area. Regarding the suggestion for lines 306-309, the purpose of the introductory paragraph in the "Results" section was to guide the reader through the structure of the results, since we are aware this is a rather complex topic with intertwined subtopics leading to a complex paper structure. The aim was to provide clarity for the readers.

F. *I have another few questions for the authors:*
Regarding "Participant observation and informal conversations":
For the reasons that authors explain in the text, I find it a very interesting method that I, personally, had not known before.
Perhaps the writing should reflect as weaknesses or strengths of the study a summary of the answers to the following questions:
-What validity, as data to analyze, do the authors give to these interviews?
-Do they have some information about the reproductivity of the data obtained in this way?
-Can they refer us to some studies in this respect?
-How have they taken into account the subjectivity of the data obtained in this way?
-Do the authors consider that they are the first ones to use this method to achieve conclusions?

Response: We are very happy the reviewer finds participant observation and informal conversations (also called informal interviews) interesting and that the reviewer stated (s)he has not had previous experience of it. The questions (s)he posed are general methodological questions, the answers to which can be found in introductory qualitative research handbooks, such as the SAGE handbook of qualitative data collection [1], of qualitative methods in health research [2] or Bryman's textbook "Social Research Methods" [3], to give a few examples. It is beyond the scope of this article to explain basic qualitative research methods.

[1] Flick U. The SAGE handbook of qualitative data collection. Sage; 2017.

[2] Bourgeault I, Dingwall R, De Vries R. The SAGE handbook of qualitative methods in health research. Sage; 2010.

[3] Bryman A. Social Research Methods, 4th Edition. 4th edition. Oxford ; New York: Oxford University Press; 2012.

F. *Ethics: Oral consent was preferred for different reasons. Can they explain the method used to register the actual given oral consent?*

Response: Oral consent was approved by both ethical review boards and documented by the interviewer obtaining consent from the participants on a separate sheet of paper in the qualitative

strand and, if present, by an additional witness belonging to the local research team. It was documented on the questionnaire form in the quantitative strand

__G.__ English language: I am not qualified to rate the correctness of language use.

14. ***Reviewer #2****: Interesting piece of work which used both qualitative and quantitative research techniques to investigate epilepsy in onchocerciasis-endemic villages in Cameroon. the authors explored in-depth the different perceptions and therapeutic attitudes of people when it comes to epilepsy. Importantly, they highlight the barriers to a successful CDTI programme which is much needed to eliminate onchocerciasis and hopefully reduce the epilepsy burden.*

I commend the efforts of the authors and recommend that this work be published after addressing a few minor concerns:

- Ref 39 in the manuscript is a conference abstract, and should be replaced by the full publication: Gumisiriza N, Kaiser C, Asaba G, et al. Changes in epilepsy burden after onchocerciasis elimination in a hyperendemic focus of western Uganda: a comparison of two population-based, cross-sectional studies. Lancet Infect Dis 2020; S1473309920301225. DOI: 10.1016/S1473-3099(20)30122-5

- On the subject of onchocerciasis control being able to reduce OAE burden, an important reference I would suggest to the authors is the meta-analysis by Siewe et al: Meta-analysis of epilepsy prevalence in West Africa and its relationship with onchocerciasis endemicity and control. Int Health 2020; 12: 192–202; DOI: 10.1093/inthealth/ihaa012. This has not been cited in the manuscript.

- Line 784, page 33: At the end of that line (... prevalence have to be...), please replace "have" with "has"

Response: We thank the reviewer for these useful comments. We adapted reference n° 39 to the latest publication (which is now n°41) and added the recent publication of the meta-analysis of epilepsy prevalence in West Africa (which is now reference n°23). Due to the Citation & Bibliography software used, these changes are however not visible as tracked changes in the paper. The mistake in line 784 has been adapted accordingly.

15. ***Reviewer #3:*** *Refer to attachment*

----

16. Attachment Reviewer #3:

Following changes are recommanded:

1. *In the abstract simple sentence is advisable (L52-54)*
2. *Conclusion should be based on fact/result rather than speculation.*
3. *Sentence should be simple and small (L133-136).*
4. *Introduction lacks clear description of hypothesis and aims and objectives.*
5. *In table 4 number of total participants from each villege may be written.*
6. *Whole paper can be rewritten and prepare for publication.*

Response: We thank the reviewer for their comments and address them point-by-point hereunder:

1. We assume the reviewer was referring to this sentence: “Ivermectin uptake for onchocerciasis was generally well known and regarded, though the CDTI was characterized by structural and logistical bottlenecks undermining equal access and optimal adherence to the drug.”. We adapted the sentence to “Ivermectin uptake for onchocerciasis was generally well known and

well regarded. The CDTI faced structural and logistical bottlenecks undermining equal access and optimal adherence to the drug.” and hope it reads easier/simpler now.

2. We are not sure which part of the conclusion the reviewer considers being speculation. The conclusion synthesizes results and discussion elements, and includes some recommendations based on these. We reviewed the conclusions and made them more concise and clearer.
3. We adapted the sentence which now reads as: “Our mixed methods study aimed to provide insights into the health seeking behaviour for epilepsy, as well as into the uptake of MDA with ivermectin through the on-going CDTI in the Sanaga River Basin, the latter of which might have an important role to play in the *prevention* of OAE.”
4. The aims and objectives are described at the end of the introduction (adapted to the reviewer’s suggestion n°3 now) and in the first part of the “Methods” section (lines 174-178) where it reads: “The purpose of the qualitative study was to gain an in-depth understanding of the health seeking behaviour for epilepsy as well as perceptions and experiences related to onchocerciasis and the ivermectin distribution programme. The quantitative cross-sectional population-based survey aimed to quantify preliminary findings from the qualitative strand”. It was a descriptive study and not a hypothesis-driven one. The study hence did not follow a hypothetico-deductive methodology but a retroductive methodology ([1]) combining inductive analysis from field data and theory from existing anthropological literature as explained in the “Methods” section, lines 322-327.

   [1] Charmaz K. Constructing grounded theory. Sage; 2014.
5. In line with another reviewer’s suggestion to avoid repetition in the paper, we propose to not add the total number of participants from each village in table 4, since this data is already presented in table 2. To make this clearer in table 2 we changed the column title “respondents” to “participants”.
6. We thank the reviewer for their comment. The text was professionally edited by Lost In translation, Belgium.

----

17. Technical Check request: *We noticed that you used "unpublished data" in the manuscript, we do not allow these references. The PLOS data access policy requires that all data be either published with the manuscript or made available in a publicly accessible database. Please amend the supplementary material to include the referenced data or remove the references. Please make a note of this change in your response to reviewers.*

Response: We have modified the sentence which mentioned unpublished data in the manuscript (lines 215-217). It was not actually referring to references to be cited, but was detailing the methods used to select the villages in which the study took place. We hope that the sentence is now clearer.
