# Supplementary material for: In pursuit of a cure: The plural therapeutic landscape of onchocerciasis-associated epilepsy in Cameroon – A mixed methods study
Source: PLoS Negl Trop Dis. 2021 Feb 23;15(2):e0009206. doi: 10.1371/journal.pntd.0009206 (PMC7946181; doi:10.1371/journal.pntd.0009206)
Supplement: Attachment — Submitted filename: comments.docx [file pntd.0009206.s008.docx]

Following changes are recommanded:

1. In the abstract simple sentence is advisable (L52-54)
2. Conclusion should be based on fact/result rather than speculation.
3. Sentence should be simple and small (L133-136).
4. Introduction lacks clear description of hypothesis and aims and objectives.
5. In table 4 number of total participants from each villege may be written.
6. Whole paper can be rewritten and prepare for publication.
